# Judged and Remembered Trustworthiness of Faces Is Enhanced by Experiencing Multisensory Synchrony and Asynchrony in the Right Order

**DOI:** 10.1371/journal.pone.0145664

**Published:** 2015-12-30

**Authors:** Hugo Toscano, Thomas W. Schubert

**Affiliations:** 1 Instituto Universitário de Lisboa (ISCTE-IUL), Centro de Investigação e Intervenção Social, Lisboa, Portugal; 2 Department of Psychology, University of Oslo, Oslo, Norway; University of Tuebingen Medical School, GERMANY

## Abstract

This work builds on the *enfacement effect*. This effect occurs when experiencing a rhythmic stimulation on one’s cheek while seeing someone else’s face being touched in a synchronous way. This typically leads to cognitive and social-cognitive effects similar to self-other merging. In two studies, we demonstrate that this multisensory stimulation can change the evaluation of the other’s face. In the first study, participants judged the stranger’s face and similar faces as being more trustworthy after synchrony, but not after asynchrony. Synchrony interacted with the order of the stroking; hence trustworthiness only changed when the synchronous stimulation occurred before the asynchronous one. In the second study, a synchronous stimulation caused participants to remember the stranger’s face as more trustworthy, but again only when the synchronous stimulation came before the asynchronous one. The results of both studies show that order of stroking creates a context in which multisensory synchrony can affect the trustworthiness of faces.

## Introduction

One’s concept of one’s own body shows a surprising degree of plasticity. Humans hold a representation of their own body, a *body image*, which is updated continuously. This process is sensitive to multiple sensorial channels and kinds of stimulation [1]. One key element of stimulations that update the body image is the synchrony of various channels. For instance, some studies (for a review see [2]) showed that participants’ own conception of their body extended to include extracorporeal body-parts in their conceptualization when seen and felt stimulations were synchronous.

The rubber hand illusion is one of the main examples of the flexibility in body representation. In this illusion, the body image changes when a prosthetic hand is being brushed synchronously to a brushing felt on the actual hand. This causes the prosthetic hand to be perceived as being one’s own hand (compared to asynchronous stroking, [3–4]). Recent studies have shown that this illusion also occurs with one of the most distinguished attributes of a person’s identity, that is to say, the face. This phenomenon is called the “enfacement effect” [5–8].

Previous work has shown that such changes during bodily illusion go along with changes as to how one perceives and relates to the individual whose face or body are involved. Synchronous rhythmic stimulation can cause people not only to incorporate others’ faces at a body representation level, but also to feel a social overlap with them, and to identify with them [5]. Thus, social effects are accompanying cognitive changes and this in turn provides an interesting bridge to explain why synchrony is such a potent element in the creation and maintenance of social relations [9].

However, the literature is currently vague as to what types of process are affected by synchrony, and when synchrony actually has reliable effects. For instance, some studies suggest that the role of synchrony may be larger when the synchronous stimulation occurs before the asynchronous stimulation, whereas others are mute on the topic [10–11]. The current research aims to extend previous work and to provide new knowledge on the subject. In particular, we will investigate the role of synchronous rhythmic stimulation for face perception as well as the effects of the order of synchrony vs. asynchrony.

## The Enfacement Illusion and its Role in Social Perception

The rubber hand illusion describes the feeling of incorporating body parts that are not actually one’s own into the individual’s body image. It was first established for hands, but rubber hand-like illusions were later extended to other parts of the body including the face. In a typical paradigm, this "enfacement effect" is created through brushing the cheek of the participants while they see the face of another person being touched synchronously. This procedure recreates the experience of looking at ourselves in a mirror, although in this case our reflection is replaced by the face of another individual. The illusion was firstly reported in a study of Tsakiris [8] during which the participants were touched on the face while seeing a video of a stranger’s face being touched; participants judged their own face to resemble the stranger’s face more strongly when the touches were synchronous rather than asynchronous. This effect was found when assessing how people judged the composition of their own face using morphs of the person’s and the stranger’s face. In sum, the stranger’s face influenced the visual representation of one’s own face after synchronous stimulation.

In a similar study, Paladino et al. [5] showed that a synchronous multisensory stimulation can have effects that extend beyond the body features, and that can play a role in social perception. In this study, as in Tsakiris’ [8], participants watched a stranger’s face being touched with a paintbrush while their cheeks were stroked in a synchronous or asynchronous way. After each type of stroking (synchronous and asynchronous), participants were asked about the illusion and needed to assess “similarity” and “closeness” with the stranger. In addition, conformity to the stranger was tested. In this conformity task (Castelli, Vanzetto, Sherman, & Arcuri, 2001; Vaes, Paladino, Castelli, Leyens, & Giovanazzi, 2003) participants were shown screens containing a large number of letters. They had to guess the number of letters. In addition, they were shown a number on the screen and told that this number was an estimate suggested by the person that they had previously watched on the screen. Conformity corresponded to the difference between the participants’ estimate and the stranger’s alleged estimate. Participants considered the stranger as more similar and felt closer to her after a synchronous multisensory stimulation in comparison to an asynchronous one. Therefore, blurred bodily boundaries between the self and others can lead us not only to see physical resemblance, but also to feel psychologically closer and more similar. The conformity measure showed that participants aligned estimates more with the numerical anchors given by synchronously touched than asynchronously touched strangers (see also [12]). Notably, the variables investigated in these studies all consist of judgments or direct measures of *relational* processes, and did not extend to judgments of other individuals.

In order to have solid experimental power, studies of multisensory synchrony typically use within-participants designs; participants hence experience both synchronous and asynchronous stimulation with different strangers. Order is typically counterbalanced, but the effects of order are rarely reported and the sample sizes are often small. However, some recent studies have found an interaction between synchronous multisensory stimulations and the order of presented stimuli. Tajadura-Jiménez, Longo et al. [11] reported that the differences between the synchronous and asynchronous stimulations for each one of the illusion components (self-identification, similarity, and affect) were much smaller when asynchrony was experienced first, but the findings were not further discussed. In an ongoing research, Schubert et al. [10] found similar order effects when replicating the effects of synchrony on perceived entitativity (*i*.*e*. perceived extent to which persons are considered as a cohesive unit; see [13–14]). The effects of synchrony on perceived cohesiveness were larger when the synchronous condition came before the asynchronous condition rather than the other way around. These findings suggest that the order in which synchrony and asynchrony are experienced should be studied more closely.

In sum, the "enfacement illusion" leads to blurred self-other boundaries, but also to an enhancement of the social closeness with others. We feel that a stranger’s face resembles our own face more when touches are experienced in temporal and spatial synchrony, and this illusion in turns increases social bonds with the other. However, it remains unclear whether psychological processes beyond the relation itself are influenced or not. Also, there are reasons to believe that the effects of this illusion may interact with the order of stroking, that is, whether the synchronous stimulation occurs before or after the asynchronous one. Our goal here is to investigate how this synchrony as well as the order of experiences might impact individuals’ face evaluation.

## Evaluations of Others’ Faces are Transferred from the Self

People rapidly infer traits from many social dimensions (e.g [15]). Even when we do not deliberately evaluate faces, studies show that we tend to rapidly categorize them [16–17]. Trait inferences from faces can influence important decisions made in different contexts such as in the political arena (e.g. [18]) or in the judicial system [19–20].

Oosterhof and Todorov [21] unpacked the underlying dimensions of those trait judgments in a bottom-up, data driven series of studies. According to their model, faces are evaluated mainly according to two dimensions: valence, which seems to be the more important one, and dominance. In their studies, all positive judgments (e.g. trustworthy, emotionally stable) loaded positively and all negative judgments (e.g. mean, weird) loaded negatively on this dimension. They also demonstrated that the valence dimension could be approximated by judgments of trustworthiness; these can thus be seen as a proxy of general valence. Oosterhof and Todorov [21] observed that faces maximizing trustworthiness resembled facial expressions of happiness, while faces minimizing trustworthiness seemed to show anger. Hence, we can conclude that facial expressions of these emotions—or facial features resembling such expressions—are determinants of trustworthiness judgments. The question is then what else can determine trustworthiness or positivity? One known mechanism is associative learning; Jones et al. [22] have shown that composites of faces paired with neutral sounds were preferred to composites of faces paired with an aversive sound. One of the sources of such learned affective connotations is regular interactions, or what is called the “familiar face overgeneralization hypothesis”. Zebrowitz and Collins [23] claimed that individuals will consider faces as being more trustworthy if they resemble the ones of significant others, friends, or even their own face. According to these authors, an individual’s idiosyncratic face preferences are partially defined by the different people with whom one is familiar with and also by one’s own appearance

There is in fact good evidence for such transfer processes. Kraus and Chen [24] demonstrated that judgments about a close other are more likely to be transferred to a new person when their faces resembled each other. According to DeBruine [25], partners in a trust game who resembled the participants' own face more were also trusted more. Recently, Farmer, McKay and Tsakiris [26] showed the reverse causal direction: When trust was reciprocal during a trust game, participants considered the game partners as resembling themselves more.

Verosky and Todorov [27] argued that learning and similarity can form a causal chain. In their research, they showed that a general mechanism of learning based on facial similarity can account for different facial preferences. For example, faces that were similar to faces previously associated with positive behaviors were considered more trustworthy than faces similar to those previously associated with negative behaviors. Verosky and Todorov [28] showed that this process of generalization may occur in an automatic way and even when the participants are instructed not to use cues related to similarity. Thus, past social interactions can influence the evaluation of novel faces. The preference for faces that resemble one’s own face fits neatly into this model, as most people have a positive association with themselves and indeed their face.

## A Role for the Enfacement Effect in Judging Trustworthiness

We have summarized so far how synchrony leads to an enfacement effect and increases resemblance and relational bonds between self and others, and also how resemblance drives evaluating others as trustworthy. Combining these two arguments, we propose that multisensory experiences of synchrony with others can render these others more trustworthy. This would extend the effects of synchrony beyond the social relation to the field of person perception. Initial support for this idea was reported by Tajadura-Jiménez, Lorusso and Tsakiris [29], who found that participants judged another’s face as more trustworthy after being touched synchronously than after an asynchronous manipulation. However, their study was focused on other variables, and no work has yet specifically investigated the role of the "enfacement illusion" in the evaluation of faces.

In the present paper, we investigate specifically how multisensory synchrony experiences affect one’s judgments of others. We investigate not only the judgment of the synchronously stimulated others, but also of individuals that merely resemble them to various degrees (Study 1). Additionally, we investigate how the bodily illusion may alter how we recognize faces (Study 2). We combine this with a high-powered test of the effects of order which remained neglected in previous work.

## Study 1

The main goal of this study is to test whether a synchronous experience with a face will influence later judgments related to that face *and* similar faces that are resemblant. We hypothesized that synchrony affects trustworthiness, and that these effects will in turn be generalized to novel faces. Thus, we predict that (a) if participants are stroked while seeing the face of another person being stroked in a synchronous way, they would judge this synchronously stroked person as more trustworthy, and (b) that the same would also occur for faces that resemble the latter, when compared to an asynchronous stroking.

In the experiment we used the face illusion paradigm [8]. Tsakiris [8], using morphs that were composites of the participant’s face and a stranger’s face, showed that when the participants were touched in synchrony with touches applied to the stranger’s face, they perceived more similarities between them and the composite face than after being touched asynchronously. In the experiment, we used a procedure that was similar to Tsarikis’ paradigm: Another’s person face was presented on a screen and was stroked in synchrony or asynchrony with stroking applied to the participant’s own face. Afterwards, participants were asked to evaluate how trustworthy the faces appeared to be.

The faces presented for the trustworthiness ratings were both the faces seen in the videos, and morphs that combined the seen faces with new faces. The new faces were morphed with 20% or 35% of the strangers’ faces (see [27]). Thus, the resemblance of the strangers’ faces with the morphed faces was not obvious, and these morphs were in the categorical boundary of the new faces, because they had at least 65% of one of the new faces [30–31]. We expected the faces morphed with the “synchronous faces” (*i*.*e*., faces with which the participant experienced synchrony) to be judged as more trustworthy than faces morphed with “asynchronous faces” (i.e. faces seen after an experience of asynchrony). Furthermore, the effect of synchrony experience should be stronger for the 35% morphs than the 20% morphs. The transformation from natural to computer-generated faces could have led to some losses in the process of morphing, that is, the computer-generated faces were not 100% equal to the faces seen on the screen during the stroking manipulation. Therefore, the levels of morphing are probably slightly less than 100%, 35%, and 20%. This implies that the 35% morphs have more than 65% of a novel face, which makes them even more novel faces.

## Method

### Ethics statement

The Ethics Committee considered our studies and indicated that it did not require a formal review because both studies described here were run in agreement with the Ethics Guidelines issued in 2012 by the Scientific Commission (Comissão Científica) of the host institution *Centro de Investigação e Intervenção Social*, Lisboa, Portugal (CISIUL). These Ethics Guidelines provide a framework to decide whether a formal review process is necessary or not. This framework indicated that the current studies were exempt from formal ethics review because data were: 1) collected anonymously without any pressure to complete the form; 2) did not involve questions about undesirable personal characteristics; 3) did not involve participants from a population of concern; 4) did not involve deception; 5) did not involve ingesting any substances; 6) did not involve invasive measures; 7) did not collect personally identifying information (i.e. name, IDs, civic or email addresses, or images); or 8) did not collect potentially endangering information. Moreover, no false information was provided, only adults were sampled, and data were then anonymized by the Scientific Commission. The first author explained the procedure to the participants when they arrived in the lab and the participants were subsequently asked to read and sign an informed consent document.

### Participants

We collected data from 72 participants in Lisbon, Portugal. The mean age was 21.10 years old, SD = 2.35.All of them were White and native Portuguese speakers; 61 of them were female. The participants were from various faculties of the host institution. All received a 5 € voucher for their participation.

### Materials, procedure, and design

We created 4 videos of individuals to be displayed during the brushing task; we will use the term “strangers” in the following (Female 1, Female 2, Male 1, Male 2; Age range: 21–25 years). All strangers were also White. The faces were pretested for trustworthiness. The differences between the male strangers’ faces were not significant, p > .05; female strangers’ faces also did not show significant differences, p > .05. In addition, we took photos of the strangers’ faces.

**Fig 1:**
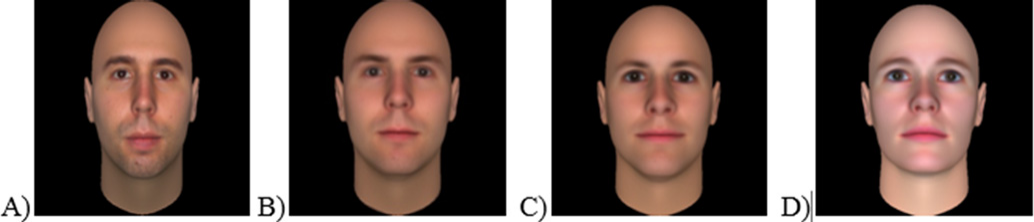
Examples of the faces judged in Study 1 (consent from the person depicted in Fig 1 A was obtained for publication of these images) A) Computerized version of the stranger’s face; B) 35% morph of the stranger’s face; C) 20% morph of the stranger’s face; D) filler face.

Because we wanted to include the actually seen faces in the trustworthiness judgment task, but did not want to have a visible difference between these faces and the morphs, we transformed the images to make them technically similar to the morphs. In particular, we created 3D models of the strangers’ faces with the software Facegen [32]. From these 3D models, we then rendered 2D images. These images, and not the original photos, were shown to participants during the trustworthiness judgment task. Thus, each stranger face that was seen in the video was judged as a computer-generated face. One of the limitations of this method might be related to the fact that while the strangers’ faces during the trustworthiness judgments were computer-generated faces and bald, they were seen in the videos with hair and as natural faces. This might cause some issues, but it enables us to take into account only the structure of the face and not other features such as the hair. Then, we created 80 morphs for each one of the four strangers’ faces with the software Morpheus Photo Mixer (http://www.morpheussoftware.net). The starting faces of these morphs were randomly taken from the Todorov Database which were also generated using Facegen (tlab.princeton.edu/). Moreover, we used filler faces (20 for each one of the strangers), see **Fig 1**.

During the experiment, a video of a stranger’s face being stroked on the cheek was shown on a screen in front of the participant. While the participant was seeing the video, her or his own cheek was stroked in synchrony or in asynchrony with the video. The videos were five minutes in duration: one minute showing the face without it being stroked, three minutes of stroking, and one more minute without stroking. Each participant saw two movies of two different strangers, and synchrony was manipulated within participant. Both strangers had the same gender as the participant. For instance, when a female participant first experienced synchrony with the “female 1” video, in the second movie she was stroked in asynchrony with the “female 2” video. Order of synchrony vs. asynchrony and assignment of the two identities to these conditions were counterbalanced. After each stroking of the face, participants evaluated faces.

#### Face evaluation

In the evaluation phase of the experiment, participants were told that we were interested in first impressions of faces, and that there were no right or wrong answers. We asked the participants to judge faces on the dimension of trustworthiness. Each of the four video faces was morphed with 40 novel computerized faces at two different levels of morphing (with 20% and 35% of the strangers’ faces—80% and 65% of novel faces respectively). Half of the novel faces were shown at the 20% morphing level and the other half at the 35% level, and these were counterbalanced across participants. The strangers’ faces seen on the screen were also evaluated (after being processed by Facegen, as described above). Thus, participants saw three types of pictures: morphs containing 20% of the original faces, morphs containing 35% of the original faces, and original faces processed through Facegen (which we will call 100% morphs).

Each trial started with a 1000-millisecond fixation cross. The face remained on the screen until the participant responded using the number keys from 1 (not at all trustworthy) to 9 (extremely trustworthy). Each participant rated all faces twice and their order was randomized.

#### Enfacement questionnaire

Besides the ratings of faces, we used a face illusion questionnaire to explore which of the variables of the illusion could mediate the ratings of faces. After each video, participants rated their sense of *ownership* (e.g., “It felt as if my face was turning into the face in the video,” 4 items), *agency* (e.g., “Sometimes I had the impression that if I had moved my eyes, the eyes of the person in the video would have moved too,” 3 items), and *location* (e.g., “It seemed as if the touch I felt was caused by the paintbrush touching the face in the movie,” 3 items; [33]).

#### Physical resemblance

Participants judged how much the stranger resembled their own facial features on a list of different features. We created an index of general resemblance and also separate indices of resemblance of core features (using only the responses regarding the regions of the mouth, eyes, and nose) and peripheral facial features (forehead, cheeks, chin, and face shape ratings; [5]). All responses were given on a scale from 1 (not at all similar) to 7 (completely similar).

#### Liking

Four items assessed how much the participant liked the face of the stranger (e.g., “How much did you like the face seen in the video?”, “How nice do you think the person seen in the video is?”).

#### Inclusion of the other in the self

Participants judged their relationship with the stranger seen on the video through a modified version of the Interpersonal Overlap Scale, which presents a series of pictures in which two circles increasingly overlap, and asks participants which overlap depicts their feeling of closeness [34].

In sum, the design of the study was 2 (Type of Stroking: Synchronous vs. Asynchronous, within participants) x 2 (Order of Stroking: First Synchrony vs First Asynchrony, between) x 3 (Morphing: 100% vs. 35% vs. 20% of the faces seen on the screen, within participants).

### Results

#### Trustworthiness judgments

We averaged the trustworthiness ratings according to conditions, and analyzed them in a 2 (type of stroking, within) x 3 (morphing, within) x 2 (order, between) GLM. We did not find a main effect of type of stroking, *F*(1, 70) = 1.83, *p* = .18, η_p_^2^ = .025. There was also no main effect of order of stroking, *F* < 1. We found a main effect for the type of morph, *F*(2, 140) = 88.43, *p* < .001, η_p_^2^ = .558. The 35% morphs (*M* = 4.66, *SD* = 1.14) were considered as more trustworthy than the 20% morphs (*M* = 4.55, *SD* = 1.02), *p* = .003, and than the strangers’ faces (*M* = 3.01, *SD* = 1.42), *p* < .001. The 20% morphs were also judged as more trustworthy than the strangers’ faces, *p* < .001. The main effect of morphing was the largest effect. This was not completely unexpected (see [27]). However, we would expect that the 100% morphs of the strangers’ faces would be judged as more trustworthy than the 35% and 20% morphs, but this was not the case. The main reason might be related to the faces used to create the morphs and the accompanying changes, which might have rendered the 100% morphs to some degree incongruent and odd; the starting faces selected from the Todorov Database were likely to be more trustworthy than the strangers’ faces. We recognize this as a potential limitation of Study 1.

However, we did find an interaction between the type of stroking and the order of stroking, *F*(1, 70) = 6.70, *p* = .012, η_p_^2^ = .087. When the synchronous condition came before the asynchronous one, participants rated the synchronous strangers’ faces as more trustworthy (*M* = 4.28, *SD* = 1.14) than the asynchronous strangers’ faces (*M* = 3.93, *SD* = 1.08), *F*(1,70) = 7.76, *p* = .007, η_p_^2^ = .100). In comparison, when asynchronous stroking came before synchronous stimulation, there was no difference between the evaluation of the trustworthiness of the synchronous strangers’ faces (*M* = 3.99, *SD* = 0.96) and the asynchronous strangers’ faces (*M* = 4.10, *SD* = 1.10), *F* < 1.

The interaction between the order of stroking and morphing was not significant, *F* < 1. We also did not find an interaction between morphing and the type of stroking, *F* < 1.

There was no significant three-way interaction between the order of stroking, the type of stroking and morphing, *F*(2, 140) = 2.25, *p* = .109, η_p_^2^ = .031. However, given that we did not expect a reversal of the effects in the condition where asynchronous stimulation came first, the contrast underlying that test is not really appropriate. We thus checked effects for the three levels of morphing separately. When synchrony was the first condition experienced, the synchronous strangers’ faces (100%) were evaluated as more trustworthy (*M* = 3.44, *SD* = 2.06) than the asynchronous strangers’ faces (*M* = 2.86, *SD* = 1.69), *F*(1,70) = 4.78, *p* = .032, η_p_^2^ = .064. Similarly, when synchrony came first, the morphs containing 35% synchronous strangers’ faces were evaluated as significantly more trustworthy (*M* = 4.81, *SD* = 1.10) than the morphs containing 35% of the asynchronous strangers’ faces (*M* = 4.49, *SD* = 1.19), *F*(1,70) = 7.87, *p* = .007, η_p_^2^ = .101. For the 20% morphs there were no significant differences between synchronous (*M* = 4.58, *SD* = 1.08) and asynchronous stroking (*M* = 4.45, *SD* = 1.01), *F*(1,70) = 1.34, *p* = .252, η_p_^2^ = .019, even when synchrony came first.

**Fig 2:**
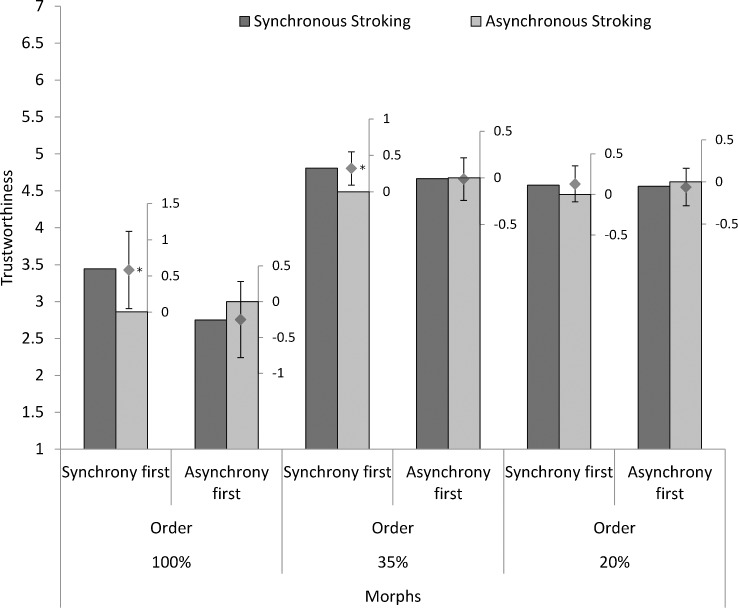
Means and differences with 95% Confidence Intervals of trustworthiness judgments according to type of stroking (synchrony vs asynchrony), order (Synchrony first vs Asynchrony first) and Morphing (100% vs 35% vs 20%). The bars represent the means of every condition. The within subject differences due to synchrony are visualized with floating scales together with their confidence intervals (constructed following [46]). In addition, asterisks mark significant differences.

When asynchronous stroking took place before the synchronous manipulation, there were no significant differences between synchronous strangers’ faces (*M* = 2.75, *SD* = 1.20) and asynchronous strangers’ faces (*M* = 3.00, *SD* = 1.43), *F* < 1. The same also occurred for the 35% morphs (Synchrony: *M* = 4.66, *SD* = 1.21; Asynchrony: *M* = 4.67, *SD* = 1.30), *F* < 1, and 20% morphs (Synchrony: *M* = 4.56, *SD* = 1.09; Asynchrony: *M* = 4.62, *SD* = 1.13), *F* < 1. **Fig 2**summarizes the results.

#### Illusion

In the face illusion questionnaire, we found main effects of synchrony for *ownership*, *F*(1,70) = 9.81, *p* = .003, η_p_^2^ = .123, *location*, *F*(1,70) = 33.17, *p* < .001, η_p_^2^ = .321, *agency*, *F*(1,70) = 8.62, *p* = .004, η_p_^2^ = .110 and *affect*, *F*(1,70) = 5.33, *p* = .024, η_p_^2^ = .071. However, the order of stroking also influenced the results. There were interactions between the order of stroking and the type of stroking for the *ownership*, *F*(1,70) = 37.59, *p* < .001, η_p_^2^ = .349, the *location*, *F*(1,70) = 11.13, *p* = .001, η_p_^2^ = .137, the *agency*, *F*(1,70) = 9.09, *p* = .004, η_p_^2^ = .115, and the *affect*, *F*(1,70) = 10.07, *p* = .002, η_p_^2^ = .126, components.

Pairwise comparisons of the four variables related to illusion showed significant differences when the synchronous manipulation condition came first: ownership, *F*(1,70) = 42.91, *p* < .001, η_p_^2^ = .380, location, *F*(1,70) = 41.37, *p* < .001, η_p_^2^ = .371, agency, *F*(1,70) = 17.71, *p* < .001, η_p_^2^ = .202, and affect, *F*(1,70) = 41.37, *p* < .001, η_p_^2^ = .177, (for the respective means see Table 1).

**Table 1:** Illusion Measures results according to the type of stroking (Synchrony vs Asynchrony) and order (Synchrony First/ Asynchrony First) in Studies 1 and 2.

	Synchrony First					Asynchrony First				
	*Synchronous Stroking*		*Asynchronous Stroking*			*Synchronous Stroking*		*Asynchronous Stroking*		
Variable	*M*	*SD*	*M*	*SD*	*p*	*M*	*SD*	*M*	*SD*	*p*
**Study 1**										
*Ownership*	1.11	1.61	-0.63	1.78	< .001	-0.68	1.60	-0.12	1.27	.038
*Location*	0.40	1.53	-1.31	1.57	< .001	0.47	1.71	0.01	1.60	.091
*Agency*	-0.38	1.80	-1.42	1.53	< .001	-0.58	1.73	-0.57	1.74	.955
*Affect*	1.26	1.47	0.56	1.79	< .001	1.12	1.37	1.23	1.29	.543
**Study 2**										
*Ownership*	-0.85	1.64	-1.55	1.64	.052	-0.85	1.90	-0.85	1.47	.987
*Location*	0.33	1.80	-1.53	1.61	< .001	0.29	1.87	-0.43	1.73	.073
*Agency*	-0.84	1.79	-1.66	1.34	.027	-0.71	1.64	-0.88	1.62	.638
*Affect*	0.63	1.59	0.32	1.61	.194	0.98	2.03	0.88	1.95	.700

In contrast, when asynchrony came first, a significant difference was only found for the *ownership* variable, *F*(1,70) = 4.50, *p* = .038, η_p_^2^ = .060. Note that the effect on ownership shows a different pattern than what was expected because it was stronger after the asynchronous stimulation than after the synchronous one. Moreover, there was a marginal difference in *location*, *F*(1,70) = 2.93, *p* = .091, η_p_^2^ = .040. In contrast, *a*gency, *F*(1,70) = 0.003, *p* = .955, η_p_^2^ < .001, and affect, *F* < 1, did not show any significant differences.

#### Physical resemblance

The variables of physical resemblance did not show any significant difference. Thus, the participants did not see themselves as more similar to the strangers’ faces when synchronously stroked (*M* = 3.22, *SD* = 1.21) compared to when asynchronously stroked (*M* = 3.19, *SD* = 1.28), *F* < 1. In addition, the resemblance of the core features did not show any difference between the synchronous (*M* = 2.93, *SD* = 1.34) and the asynchronous (*M* = 2.89, *SD* = 1.27) stimulations, *F* < 1. The pattern was the same for the synchronous (*M* = 3.12, *SD* = 1.26) and the asynchronous (*M* = 3.19, *SD* = 1.41) manipulations in regards to the resemblance of the peripheral facial features, *F* < 1. Yet when the order of the conditions was taken into account, there was an interaction between the order of stroking and the type of stroking for the general physical resemblance, *F*(1, 70) = 4.78, *p* = .032, η_p_^2^ = .064, and the peripheral facial features, *F*(1, 70) = 4.06, *p* = .048, η_p_^2^ = .055. However, the pairwise comparisons only showed a marginal difference when synchrony came first for the general resemblance variable (*M*_Synchrony_ = 3.14, *SD* = 1.32, *M*_Asynchrony_ = 2.89, *SD* = 1.46), *F*(1, 70) = 3.08, *p* = .084, η_p_^2^ = .042. For the resemblance of the peripheral features, we also found a marginal difference between synchronous and the asynchronous conditions, but only when asynchrony came first (*M*_Synchrony_ = 3.18, *SD* = 1.13, *M*_Asynchrony_ = 3.53, *SD* = 1.13), *F*(1, 70) = 3.24, *p* = .076, η_p_^2^ = .044. The core resemblance variable did not show any interaction with the order of the stroking (*p* = .765).

#### Liking

Participants also did not like the face more after the synchronous stimulation (*M* = 3.75, *SD* = 1.10) than after the asynchronous one (*M* = 3.86, *SD* = 1.05), *F* < 1. There was no interaction with order as to the rating of the liking of faces either, *p* = .383.

#### Inclusion of the other in the self

For the felt overlap, we found that participants did not feel more overlap on the IOS when the faces were synchronously stimulated (*M* = 2.44, *SD* = 1.14) than when asynchronously stimulated (*M* = 2.44, *SD* = 1.18), *F* < 1. There was no interaction with order of the stroking, *p* = 1.00.

## Discussion

In Study 1, we tested whether experiencing stimulation on one’s own face in synchrony with the perceived stimulation on a stranger’s face influences how trustworthy that stranger is then judged. We manipulated synchrony within participants and counterbalanced the order of synchronous vs. asynchronous stroking as it is typically done in such studies. We predicted that synchronous stimulation would lead to both an enfacement illusion and enhanced judgments of trustworthiness. We did find this difference, but *only* when synchronous stimulations came before asynchronous stimulations. For the enfacement illusion, both main effects of synchrony and an interaction with the order were observed; for trustworthiness, only the interaction (synchrony x order) was present. Synchrony only increased trustworthiness for the original version of strangers’ faces and morphs to which those faces contributed to 35% of the constitution. As expected, no difference was found if the stimulated strangers’ faces contributed to only 20% of the composition of the morphs even when synchrony came first (see [27]). In contrast, when the synchronous stimulation *followed* the asynchronous stimulation, we found neither a reliable enfacement effect nor changes in trustworthiness.

Even though the within-subject manipulation of synchrony is a standard practice, previous studies rarely reported tests of order effects, possibly due to small cell sizes (e.g., [5]). To our knowledge it has never been systematically investigated in regards to multisensory synchrony, but it is in line with some recent studies that also observed it [10–11]. This suggests that it may not be a false positive, but indicate an overlooked moderator.

Importantly, the effect was not reversed but simply null when asynchrony came first. Such interactions might not have been noted in earlier work for a number of reasons such as the fact that sample sizes are often small, depriving interaction tests of statistical power. In previous work using different variables, the effects in the synchrony-first condition might have been larger than the one we tested here, lifting the main effect to significance. Similarly, we were able to assess an interaction effect for both the enfacement illusion and trustworthiness; this was present even if the main effect for the enfacement illusion variable was significant whereas the main effect for trustworthiness was not. In addition, the contrasting pattern underlying the typically tested interaction (i.e., weights of +1–1–1–1) is not the pattern that we observe (which is instead +3–1–1–1), and thus has reduced statistical power to detect this moderation.

Notably, we did replicate the enfacement effect indexed by the report of an illusion. However, we did not replicate the previously found effects on judged resemblance, liking, and the overlap measure even when synchrony was experienced first. We will come back to this in the General Discussion.

### Study 2

Study 2 aims to conceptually replicate Study 1. We used a very similar design, but measured a different dependent variable that is more indirect. Specifically, instead of having participants rate the trustworthiness of a face, we presented them the face they saw in the video together with morphs of that face. These morphs contained either various degrees of a very trustworthy face, or various degrees of an untrustworthy face. Participants had to select which of the presented faces they saw during the video. Thus, we tested whether they would distort their memory of the synchronously or asynchronously stimulated face either towards trustworthy or untrustworthy appearance. To follow up on the interaction with order in Study 1, we again counterbalanced order.

## Method

### Participants

We had 36 participants. They were all White women, the mean age was 21.25 years old, *SD* = 3.07. The participants were recruited from various faculties of the Lisbon University and the Lisbon University Institute. Every participant received a 5 € voucher or a university credit for their participation. The low number of participants limit the interpretations derived from Study 2. Given the size of our sample, we tested the assumptions for the GLM in relation to the trustworthiness judgments. These judgments had a normal distribution (ps > .05) after asynchrony independently of the order of stroking, and after synchrony when the first stroking was asynchronous. However, the distribution was not normal (p < .05) after synchrony when the first stroking was synchronous. Nonetheless, trustworthy judgments had homogeneous variances, ps > .05. Therefore, our data do not violate the assumption of homogeneity of variance. Also, it only violates the assumption of normality in one of four conditions. Given that the statistical power of a GLM is not greatly affected by non-normality [35] and that type-I errors are well controlled in a GLM under these conditions [36], we concluded that our data analysis should be analyzed with GLM.

### Materials, procedure, and design

Overall, the procedure was the same as in Study 1. We used two videos of female strangers (Female 1, Female 2) because we only sampled female participants. We also took photos of the strangers’ faces. As in Study 1, we stroked the participants' cheek while they were seeing the videos (same duration as in Study 1). Each participant saw both movies, and their faces were stroked in a synchronous or an asynchronous manner. Again, order of synchrony vs. asynchrony was counterbalanced. As in Study 1, we used the questionnaires of the enfacement illusion, physical resemblance, liking, and inclusion of the other in the self.

**Fig 3:**
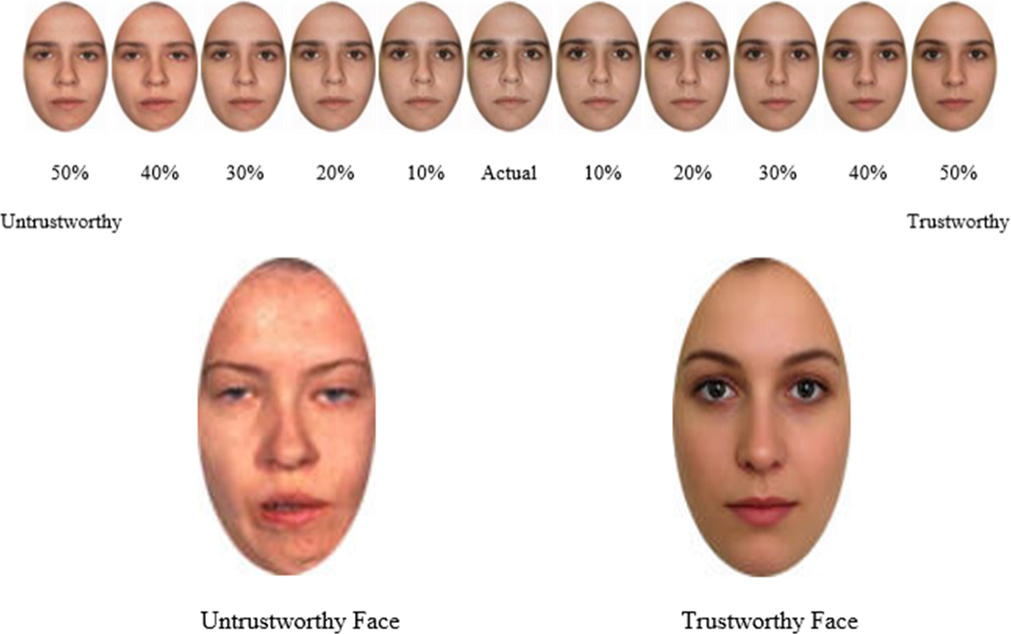
Example of the Faces and Morphs used in Study 2 (consent from the person depicted was obtained for publication of these images)

**Face selection.** After each video, the participants were presented a lineup of pictures that contained 11 faces in random order (see [37]) and were then asked to identify the face they saw previously on the screen. One of the faces was the actual face seen during stroking, five were morphed with a trustworthy face (up to 50%), and five were morphs with an untrustworthy face (up to 50%) (see **Fig 3**). These two faces were pretested for trustworthiness. The trustworthy face was judged as significantly more trustworthy, p < .05.

The study thus had a 2 (Type of Stroking: Synchronous vs. Asynchronous, within) x 2 (Order: First Synchrony vs. First Asynchrony, between) design.

## Results

### Trustworthiness measure

The main dependent variable was whether participants would chose a more trustworthy or more untrustworthy morph of the face they saw during stimulation as the face they remembered. This variable could theoretically vary from -5 to +5; a value of “0” means that the correct face was chosen, a positive value indicates that a more trustworthy face was chosen, and a negative value indicates that a more untrustworthy face was chosen. This variable was submitted to a 2 (Stroking type) x 2 (Order) GLM.

There was no main effect of stroking type; participants did not selected a more trustworthy face after a synchronous stimulation (*M* = 0.40, *SD* = 1.30) compared to asynchronous stimulation (*M* = -0.16, *SD* = 2.20), *F*(1, 34) = 1.52, *p* = .23, η_p_^2^ = .043. There was also no main effect of order; equally trustworthy faces were selected when synchronous stimulation (*M* = -0.03, *SD* = 1.12) or asynchronous stimulation (*M* = 0.27, *SD* = 1.12) came first, *F* < 1.

**Fig 4:**
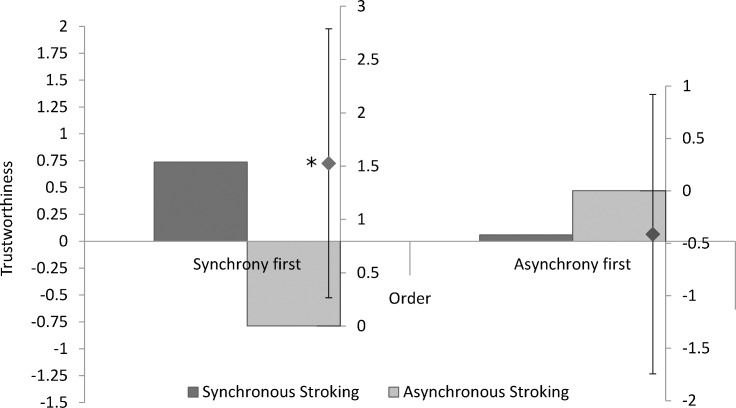
Means and differences of Trustworthiness recognition according to type of stroking (synchrony vs asynchrony) and order (Synchrony first vs Asynchrony first). The bars represent the means. The within subject differences due to synchrony are visualized with floating scales together with their confidence intervals (constructed following [46]). In addition, asterisks mark significant differences.

As in Study 1, we observed an interaction between the type of stroking and the order of stroking, *F*(1, 34) = 4.60, *p* = .039, η_p_^2^ = .119. When the first stroking was synchronous, participants selected a more trustworthy face after synchrony (*M* = 0.74, *SD* = 1.27) than after asynchrony (*M* = -0.79, *SD* = 2.14), *p* = .019. However, when the asynchronous stimulation came first, there was no significant difference between synchronous (*M* = 0.06, *SD* = 1.27) and asynchronous stimulations (*M* = 0.47, *SD* = 2.14), *p* = .53. This pattern thus resembles the one found in Study 1 (see **Fig 4**).

### Illusion measure results

We found a main effect of synchrony for *location*, *F*(1, 34) = 23.02, *p* < .001, η_p_^2^ = .404. The location illusion was significantly larger after synchrony (*M* = 0.31, *SD* = 1.80) than after an asynchronous (*M* = -0.98, *SD* = 1.73) stroking. We did not find main effects of type of stroking for *ownership*, (*M*_Synchrony_ = -0.85, *SD* = 1.74, *M*_Asynchrony_ = -1.22, *SD* = 1.58), *F*(1, 34) = 1.95, *p* = .171, η_p_^2^ = .054, *agency*, (*M*_Synchrony_ = -0.78, *SD* = 1.70, *M*_Asynchrony_ = -1.29, *SD* = 1.51), *F*(1, 34) = 3.75, *p* = .061, η_p_^2^ = .099, and *affect*, (*M*_Synchrony_ = .80, *SD* = 1.80, *M*_Asynchrony_ = 0.58, *SD* = 1.78), *F*(1, 34) = 1.42, *p* = .241, η_p_^2^ = .040.

The order of stroking again moderated the results. There was an interaction between the order of stroking and the type of stroking for *location*, *F*(1, 34) = 4.43, *p* = .043, η_p_^2^ = .115. Pairwise comparisons showed that the differences were significant regarding the location illusion, *F*(1, 34) = 25.23, *p* < .001, η_p_^2^ = .426, only when synchrony came first; when asynchrony came first, only a marginal difference was found, *F*(1, 34) = 3.44, *p* = .073, η_p_^2^ = .092. There was no interaction between the order of stroking and the type of stroking for *ownership*, *F*(1, 34) = 1.89, *p* = .178, η_p_^2^ = .053, *agency*, *F*(1, 34) = 1.83, *p* = .221, η_p_^2^ = .044, or *affect*, *F* < 1. However, the pairwise comparisons showed that when the synchronous manipulation came first, *agency*, *F*(1, 34) *=* 5.37, *p* = .027, η_p_^2^ = .136, showed significant differences, whereas *affect* did not, *F*(1, 34) *=* 1.75, *p* = .194, η_p_^2^ = .049. As for *ownership*, *F*(1, 34) *=* 4.07, *p* = .052, η_p_^2^ = .107, the difference was marginal when synchrony came first.

When asynchrony came first, we only found a marginal difference for location, *F*(1, 34) *=* 3.45, *p* = .073, η_p_^2^ = .092. We did not find differences for *agency*, *ownership*, and *affect*, *all Fs* < 1 (see Table 1).

### Physical resemblance results

The type of stroking did not have an effect on the physical resemblance variables, *F*s < 1. There was also no interaction of synchrony and order on these variables for the general physical resemblance variable, *F*(1, 34) *=* 2.08, *p* = .158, η_p_^2^ = .058, and *F*s < 1 for both the peripheral and core facial features variables.

### Liking and inclusion of the other in the self results

The type of multisensory stimulation also did not have a significant effect on the liking variable, *F* < 1. The interaction with order was also not significant, *F*(1, 34) *=* 1.98, *p* = .168, η_p_^2^ = .055. We found the same pattern in the overlap variable for the type of stroking, *F*(1, 34) *=* 1.58, *p* = .218, η_p_^2^ = .044, and when testing for an interaction with order, *F* < 1.

## Discussion

The central prediction of Study 2 was confirmed: Synchrony led to a biased recognition of faces, such that the face perceived during the stimulation was remembered as looking more trustworthy, but only when this synchronous stimulation came first and not when it came second. This suggests that the order effect found in Study 1 was not a false positive, and order deserves a more comprehensive investigation in future studies. Moreover, when the synchronous stimulation came first, there were more differences across variables related to the face illusion than when the asynchronous stimulation came first. This is an interesting result that would deserve more attention in future research.

## General Discussion

Recent studies showed that a synchronous multisensory stimulation can have an effect on social judgments by blurring the self-other boundaries and by establishing a social relation [5, 12]. In the present research we investigated whether such effects extend to formation of impressions and memories related to another person’s face. We found that under specific conditions, this could indeed be the case; sharing a synchronous stimulation (compared to shared asynchronous stimulation) can lead participants to judge the other’s face, and faces similar to the latter, as more trustworthy (Study 1), and remember the other’s face as more trustworthy than it really is (Study 2). Thus, the effects of synchrony can extend beyond the bond felt towards the synchronously stimulated person as well as the changes to the self-concept, and actually can render one’s impression and memory of a synchronously stimulated other more trustworthy (see also [29]).

Our data from Study 1 show that if synchrony has an effect on the impression of a stranger, this effect is also generalized to similar faces. As the data from the series of 35% morphs demonstrate, these morphs were also judged as more trustworthy when the participants were in synchrony with the stranger’s face that was morphed. Previous work by Zebrowitz [38] has shown that we tend to prefer individuals that resemble people whom we consider important in our lives. These results show that an embodied manipulation (i.e. a synchronic rhythmic movement) can change the judgment of faces and, consequently, drive our face preferences towards more similar ones.

However, it is important to note that this effect only occurred when the synchronous stimulation was experienced before the asynchronous stimulation. In Study 1, when the asynchronous condition was experienced first, the following synchronous rhythmic movement did not influence the judgments of how trustworthy faces appeared to be. The lack of effects on social variables in this order condition was moreover accompanied by a smaller enfacement illusion. Similarly, in Study 2, the effect of synchrony on recognition was only significant when synchrony came first. Hence, in both studies, we found a moderation of the effect of synchrony by the order in which synchrony and asynchrony were experienced. In studies of multisensory synchrony, such interactions have only previously been reported by Tajadura-Jiménez, Longo et al. [11], and were only briefly mentioned. To our knowledge, earlier studies using this design never reported such interactions, nor were the results of their tests discussed; this is possibly due to the much smaller cell sizes at hand that thus led to a lack of statistical power, or because the interaction contrast did not fit the pattern, resulting in low statistical power of the test. In ongoing work, Schubert and collaborators [10] found a similar effect of order using a different paradigm: In that research, it was observed that synchrony of two strangers had a larger impact on their perceived cohesiveness when it came before asynchrony.

One possible explanation for the interaction pattern is that synchrony is an unexpected cue in the absence of a pre-established relationship. When asynchrony comes first, attention is directed towards other cues, and judgments are tuned to this condition; hence if synchrony comes second, this feature is not taken into account anymore. However, if synchrony comes first, it captures all attention, and the lack of synchrony in the subsequent experience leads to a decrease of judged closeness. In other words, it seems that the absence of synchrony in the first trial is not diagnostic, while the disappearance of synchrony in the second trial is highly noticed by an individual. In contrast, adding synchrony in the second trial is similarly non-diagnostic, possibly because a judgment of the situation has already been formed. We ran an additional study (see method and results in **S1 File**) using the same method as in Study 2, but preceded by strong primes of social relations (friendship and authorities). No significant main effects or interactions were observed, but the pattern of order effects differed: The differences between synchrony and asynchrony were slightly larger when the asynchronous condition came first. It is possible that just as order serves to establish a context in which (a)synchrony has or does not have an effect on social variables, so can a previous context (here a prime) already establish that context beforehand.

We believe that such order effects may have been overlooked in previous work because they are probably a lot less common in non-social contexts in which these bodily illusions have first been observed and investigated. We also believe that it is not surprising that the order component, which creates a social context, plays a role when social variables such as the feeling of trustworthiness are investigated. Nevertheless, we are of course aware that these effects need to be further investigated. As a first step, future studies in this field should routinely test for these interactions and report their size even if they are not significant, which could help eventual analysis to estimate their size more precisely.

One of the surprising findings was the absence of an effect on the assessment of physical resemblance and the feeling of proximity towards the stranger after a synchronous stroking compared to an asynchronous one. Perhaps this can be explained by the fact that the face is the most important feature of our appearance and plays a major role in our self-identity; given the uniqueness of the face in our mental, some studies (e.g. [6]) suggest that it is harder for the participants to integrate in their facial representation the strangers’ facial features. Therefore, the level of malleability in the enfacement illusion is smaller compared to other types of illusions (e.g., the classic rubber-hand illusion). Consequently, it is possible that in our studies the strength of the enfacement illusion was not large enough to lead the participants to feel more similar and closer towards the stranger. It is also possible that the additional lengthy assessments taken between the synchrony manipulation and the relational measures attenuated the effect of the latter. Nevertheless, these results suggest that previous results on the relational measures should be carefully checked in meta-analysis and ideally be replicated.

Another potential limitation of our study was that we only recruited female participants in Study 2. However, previous studies did not reveal gender differences in the enfacement illusion or its downstream consequences (see [11]).

In sum, we find that the effects of experiencing multimodal synchrony with another person, and the accompanying enfacement illusion of merging bodily representations, can impact how trustworthy the person is then judged to be and how he or she is remembered to look like. The effects go even beyond the specific face one saw, and also affect faces that look alike. However, these effects are highly dependent on the order of the manipulations.

Previous work showed that synchrony can lead to the formation of a common identity, increase cooperation, and influence social perception [5, 9, 39, 40]. The results that we reported here were found with a manipulation of multisensory synchrony, not synchronous behavior: Our participants did not move themselves. We argue that our results are nevertheless informative for the effects of synchrony because behavioral synchrony contains the features of our multisensory synchrony condition, but in addition, the sensory experiences are an outcome of one’s own actions and intentions. Imagine soldiers marching in synchrony: Multisensory synchrony is given in the synchrony of feeling one’s own feet striking the ground and seeing other’s feet striking the ground simultaneously. In addition, the movement of the other’s feet are in synchrony with one’s own movements, and both follow one’s intentions to move, which will even enhance the illusion of linked bodies [41].

Interestingly, similar findings as in the synchrony literature have been documented for mimicry. Mimicry, the imitation of other people’s behavior, is similar to synchrony because to imitate others we have to match their gestures, postures, facial expressions, etc.–but instead of synchronously, the mimicry can follow with a temporal delay. Mimicry also has been found to increase rapport, affiliation and prosocial behavior [42]. Given the similarity between synchrony and mimicry, one might wonder whether our results are informative about mimicry as well. With respect to the effect on trustworthiness, our current model and results only allow cautious generalization. We derived our hypothesis from the link between the enfacement effect, resemblance, and trustworthiness. It is unclear whether a similar chain can connect experienced mimicry, similarity, and trustworthiness. Similarity is presumably a less strong glue than bodily resemblance, and experienced mimicry is a less attention-grabbing experience than the enfacement effect. Nevertheless, it is possible that the positivity of experiencing behavioral mimicry leads to enhanced judgment of trustworthiness and associated changes in face representations. In addition, the type of mimicry will matter. For instance, mimicry of facial expressions of emotions is determined by many factors besides affiliative tendencies, and interacts strongly with the situation and meaning of the expression [43, 44]. Other types of mimicry, for instance pupil mimicry, have also been linked to social interactions involving trust decision [45]. However, this type of mimicry does not involve any representation of the body at all, and is thus very unlikely to influence trustworthiness via the process hypothesized here.

However, our results on the importance of order could be crucial for studies of mimicry as well. Just as the meaning of (a)synchrony seems to change depending on whether the same situation was experienced differently before, the meaning of (not) being mimicked might change depending on whether the previous situation featured the same experience. Whenever mimicry is manipulated within participants, the order of experiences should be considered and modeled in the analyses, as it could moderate the results and thereby distort analyses if not considered.

Our findings extend this notion by suggesting that processes of synchronous multisensory stimulation can lead in some cases to change in face evaluation and memory. For instance, watching oneself in the mirror essentially resembles a multisensory synchrony experience with the face seen in the mirror–one’s own. This experience may explain partly why we like our own faces. Furthermore, experiencing behavioral synchrony with close others is very likely, and this could lead to cascading effects on judged trustworthiness of similar faces in the manner found here. Thus, multisensory synchrony may actually influence face evaluation processes in daily life.

Our observations in regards to the importance of the order of experienced synchrony and asynchrony may be crucial to understand how social rituals use synchrony to influence communal relations. Synchronized routines that intend to increase solidarity, coordination, or unity in a group (e.g., a military parade) may be more effective if they start by showing a synchronous behavior because it immediately draws attention and assigns meaning to the routine. In contrast, if synchrony emerges only over time, it may need additional factors to be deemed meaningful.

## Supporting Information

S1 File Method. (DOCX)
