# Supplementary material for: Judged and Remembered Trustworthiness of Faces Is Enhanced by Experiencing Multisensory Synchrony and Asynchrony in the Right Order
Source: PLoS One. 2015 Dec 30;10(12):e0145664. doi: 10.1371/journal.pone.0145664 (PMC4696736; doi:10.1371/journal.pone.0145664)
Supplement: S1 File — (DOCX) [file pone.0145664.s001.docx]

**Supporting Information**

**S1 Method**

## Method

**Participants**

We collected data from 80 female participants (*M* = 22.31, *SD* = 3.26) from the Lisbon University Institute, Portugal. All received a 5 € voucher for their participation.

***Materials, procedure, and design***

These were similar to Study 2. However, before seeing the videos, participants were primed either with authority or with friendship. In these priming procedures, we asked participants to think about and define a typical person who has authority (vs. a friend), and to write down the five most important aspects that can describe an authority (vs. a friend). Thus, the design was a 2 (Type of Stroking: Synchronous vs. Asynchronous) X 2 (Order: First Synchrony vs First Asynchrony) within participants x 2 (Prime: Friendship vs Authority) between participants.

**Results**

Participants did not recognize the face as more trustworthy after synchrony (*M* = 1.07, *SD* = 1.92) than after asynchrony (*M* = 0.63, *SD* = 1.89), *F*(1, 76) = 2.42, *p* = .12, η_p_^2^ = .031. We did not find an order effect. The same level of trustworthiness was selected whether the synchronous stimulation (*M* = 0.98, *SD* = 1.43) or asynchronous stimulation (*M* = 0.72, *SD* = 1.43) came first, *F*(1, 76) = 0.65, *p* = .42, η_p_^2^ = .009.

The interaction between type of stroking and order of stroking was not significant, *F*(1, 76) = 2.56, *p* = .114, η_p_^2^ = .033. The pattern was different from Study 2. When synchronous stimulation came first, choices were virtually identical after synchrony (*M* = 0.97, *SD* = 1.95) or asynchrony (*M* = 0.98, *SD* = 1.88), *p* = .975. When asynchronous stimulation came first, there was a significant difference between the synchronous (*M* = 1.17, *SD* = 1.95) and asynchronous stimulations (*M* = 0.27, *SD* = 1.88), *p* = .029.

There was no interaction between type of stroking, order of stroking and prime, *F*(1, 76) = 0.73, *p* = .787, η_p_^2^ = .001.
